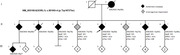# A novel stop‐gain SORL1 mutation from Amerindian background in a Peruvian family with Alzheimer’s Disease of the PeADI Study

**DOI:** 10.1002/alz.090818

**Published:** 2025-01-03

**Authors:** Mario Cornejo‐Olivas, Anthony J. Griswold, Ana Saldarriaga‐Mayo, Pedro R. Mena, Richard S. Rodriguez, Larry D. Adams, Patrice L. Whitehead, Rosario Isasi, Maryenela Illanes‐Manrique, Elison Sarapura‐Castro, Farid Rajabli, Katalina F McInerney, Karina Milla‐Neyra, Carla Manrique‐Enciso, Gary W Beecham, Sheila Castro‐Suarez, Peter St George‐Hyslop, Ismael Araujo‐Aliaga, Michael L. Cuccaro, Jeffery M. Vance, Margaret A Pericak‐Vance

**Affiliations:** ^1^ Neurogenetics Working Group, Universidad Científica del Sur, Lima Peru; ^2^ Neurogenetics Research Center, Instituto Nacional de Ciencias Neurológicas, Lima Peru; ^3^ John P. Hussman Institute for Human Genomics, University of Miami Miller School of Medicine, Miami, FL, USA, Miami, FL USA; ^4^ Dr. John T. Macdonald Foundation Department of Human Genetics, University of Miami Miller School of Medicine, Miami, FL USA; ^5^ John P. Hussman Institute for Human Genomics, University of Miami Miller School of Medicine, Miami, FL USA; ^6^ Atlantic Fellow for Equity in Brain Health at Global Brain Health Institute (GBHI), San Francisco, CA USA; ^7^ CBI en Demencias y Enfermedades Desmielinizantes del Sistema Nervioso, Instituto Nacional de Ciencias Neurológicas, Lima Peru; ^8^ Columbia University Irving Medical Center, New York, NY USA

## Abstract

**Background:**

Common and rare variants in *SORL1* have been associated with increased risk of Alzheimer’s disease (AD). Since 2019, we have run an international collaborative research initiative to ascertain a Peruvian cohort for Alzheimer’s disease and other related dementias for genetic studies (PeADI).

**Method:**

A Peruvian family (4 AD cases and two mild cognitive impairment (MCI) cases) was recruited through the PeADI study. All six family‐member completed a full cognitive assessment, had plasma‐based biomarkers pTau181 and Aβ42/40 measured via SIMOA chemistry on the Quanterix HD‐X, and underwent whole genome sequencing. Variants within AD risk genes as determined by the ADSP Gene Verification Committee were prioritized and variant interpretation was performed according to ACMG recommendations.

**Result:**

We identified a SORL1 c.5019G>A (p.Trp1673Ter) variant of Amerindian background in the four AD diagnosed siblings within this family. The two MCI cases did not carry the novel variant. The identified SORL1 variant corresponded to a heterozygous stop‐gain variant in exon 36 replacing tryptophan by a stop codon at position 1673 of the SORL1 protein. In‐silico analysis predicts this variant promotes nonsense‐mediated mRNA decay. This variant has not been previously reported in databases including gnomAD, LOVD and ClinVar. The 4 AD cases had on average 2.3X higher plasma pTau181 concentrations compared to the 2 MCI (2.03 ± 0.28pg/µl vs 0.88 ± 0.7pg/µl). There was no noticeable difference in the Aβ42/40 ratio. This variant is classified as likely pathogenic according to ACMG.

**Conclusion:**

We report the first Peruvian AD family carrying a likely pathogenic stop‐gain SORL1 variant within an Amerindian background region. Further cosegregation and functional assays are required to establish the risk size of this variant for AD.